# Distribution of endangered Italian gudgeon *Romanogobio benacensis* (Cypriniformes, Cyprinidae, Gobioninae) with remarks on distinguishing morphological characters

**DOI:** 10.3897/zookeys.729.20615

**Published:** 2018-01-16

**Authors:** Dušan Jelić, Mišel Jelić, Petar Žutinić, Ivana Šimunović, Primož Zupančič, Alexander M. Naseka

**Affiliations:** 1 Croatian Institute for Biodiversity, Maksimirska cesta 129/5, HR-10000 Zagreb, Croatia; 2 BIOTA j.d.o.o./Ltd, Braće Radića 128A, HR-43290 Grubišno Polje, Croatia; 3 University of Zagreb, Faculty of Science, Department of Biology, Rooseveltov trg 6, HR-10000 Zagreb, Croatia; 4 Dinaric Research Institute, Dolsko 14, SI-1262 Dol pri Ljubljani, Slovenia; 5 Faculty for Biology and Soil, Saint Petersburg State University, Universitetskaya Emb. 7/9, Saint Petersburg 199034, Russia

**Keywords:** Adriatic basin, freshwater fish, genetic barcoding, morphology, paleo-Po River, trans-Adriatic paleo-dispersal

## Abstract

Distribution data on many freshwater fish species in Croatia are scarce and species identifications are difficult, requiring further detailed studies. This paper presents a report of the Italian gudgeon *Romanogobio benacensis* from the Mirna River in the Istra Peninsula in Croatia, in the south-east from its previously known distribution range. The identification of *R. benacensis* in Croatia was supported by a morphological comparison with *R. benacensis* from Italy and Slovenia, the common gudgeon *Gobio gobio*, and the Danubian gudgeon *Gobio obtusirostris* from geographically close locations. A combination of character states (number of scales between anus and anal-fin origin, branched pectoral-fin rays, lateral-line scales, total, abdominal, and caudal vertebrae, and the size and number of lateral blotches) distinguishes *R. benacensis* from both *G. gobio* and *G. obtusirostris*. The phylogenetic analyses using mitochondrial sequences of cytochrome b gene confirmed that specimens from the Mirna River belong to *R. benacensis*. Also, Reka River system (Adriatic Sea basin) in Slovenia is inhabited by a possibly introduced Danubian gudgeon, *G. obtusirostris*, and not by *R. benacensis*.

## Introduction

The richness of Croatian freshwater ichthyofauna manifests in at least 147 native fish and lamprey species, many of which are endemic (Mrakovčić et al. 2006, Jelić et al. 2008, Jelić 2011b). The number of fish species in Croatian freshwater environments is continuously increasing as a result of new species descriptions (Zupančič and Bogutskaya 2002, Marčić et al. 2011, Bogutskaya et al. 2012) and re-discovery of the previously described species (Jelić 2011a, Jelić and Jelić 2015). For example, *Telestes miloradi* Bogutskaya, Zupančič, Bogut & Naseka, 2012, an endemic species whose description is based on material deposited in a museum, collected more than 100 year ago, and which had been considered extinct, was recently re-discovered in nature (Jelić and Jelić 2015).

Another example is the Italian gudgeon *Romanogobio benacensis* (Pollini, 1816), which was firstly recorded in Croatia in 2011 (Jelić 2011a). This cyprinid fish species, belonging to Palearctic subfamily Gobioninae, was originally described as *Cyprinus benacensis* from specimens collected in Lake Garda in the Po drainage (Italy). Later, the Italian gudgeon was considered a subspecies of the common gudgeon *Gobio gobio* (Linnaeus) (Bianco and Taraborelli 1984, Bianco 1988, Pizzul et al. 1993, Bănărescu et al. 1999) or a valid species *Gobio benacensis* (Kottelat 1997, Bianco and Ketmaier 2001, 2005, Kottelat and Persat 2005). Currently, the species is assigned to *Romanogobio* Bănărescu (Kottelat and Freyhof 2007, Zupančič et al. 2008). Systematic position of the Italian gudgeon within *Romanogobio* is supported by phylogenetic reconstructions using mitochondrial DNA (mtDNA) sequences of genes coding for cytochrome b (cytb) (Bianco and Ketmaier 2005) and the cytochrome c oxidase subunit I (COI) (Geiger et al. 2014). However, the basal node in the *Romanogobio* clade which shows divergence between *R. benacensis* and the remaining subclades was not supported, thus preventing Bianco and Ketmaier (2005) to consider *Romanogobio* as a supported clade in comparison with the *Gobio* Cuvier clade.

Based on some diagnostic morphological characters, the Italian gudgeon is considered more similar to *Gobio* than to *Romanogobio* (Bianco and Ketmaier 2005, Kottelat and Freyhof 2007, Bianco 2014). According to Bianco and Ketmaier (2005), a single character discriminating *R. benacensis* and *G. gobio* was the number of scales between the anus and the anal-fin origin, 2–4 in the former species and 4–8 in the latter. Kottelat and Freyhof (2007) added that in *R. benacensis* the distance between the anus and the anal-fin origin is distinctly smaller than the eye diameter, while in *Gobio* it is equal to or greater than the eye diameter. Further, in *R. benacensis* the scales on the abdomen extend only to a point between the pectoral and pelvic-fin bases, while in *Gobio* they sometimes extend to a level of the posterior end of the pectoral-fin base. However, systematic position of *R. benacensis* is controversial, since some of the morphological characters of *R. benacensis* do correspond to those diagnostic of the genus *Gobio*, while others are typical for *Romanogobio* (Kottelat and Freyhof 2007).

For nearly two centuries after its description, *R. benacensis* was considered an Italian endemic species, native in the Padano-Venetian district from the Isonzo River in the north to the Marecchia River in the south (e.g. Bianco and Taraborelli 1986, Bianco 1991, Bianco and Ketmaier 2005). First finding of Italian gudgeon outside of Italian territory was reported by Povž et al. (2005) in the lower reaches of Vipava River in the Soča (Isonzo) drainage (Slovenia). Crivelli (2006) cited a personal communication by M. Povž that *R. benacensis* was also found in the Reka River in the Adriatic basin in Slovenia. This was published later by Zupančič (2008) and Povž et al. (2015). However, no diagnostic characters of specimens from the Reka River were given to support this identification. Kottelat and Freyhof (2007) presumed that the Italian gudgeon probably occurs elsewhere in the northern Adriatic basin. Out of the native range, the Italian gudgeon was introduced and established in the Arno, Tiber, and Ombrone rivers in central Italy (Bianco 1994, Bianco and Ketmaier 2005).

In recent years, *G. gobio* was introduced in Italy and became invasive species in river systems down to the Badolato River in the south, making a serious threat to *R. benacensis* (Bianco and Ketmaier 2005). Phylogenetic inferences on mtDNA sequences of cytb (Bianco and Ketmaier 2005) and COI (Geiger et al. 2014) genes showed that the examined specimens of *G. gobio* from the Po drainage shared identical haplotypes with *G. gobio* from the Rhône drainage. However, Bianco and Ketmaier (2005) and Bianco (2009) indicated possible introductions of Danubian *G. gobio* in Italy (which refers to the Danubian gudgeon *Gobio obtusirostris* Valenciennes according to the recent taxonomic concept). *Gobio gobio* and *G. obtusirostris* are larger-sized fishes (SL up to 125–130 mm vs. 80–110 in *R. benacensis*) and, if successfully established, they might cause a considerable decline in populations, and even extirpation, of *R. benacensis* (Bianco and Ketmaier 2005, Bianco 2009). The latter species is thought to be represented only by genetically “pure” populations in its native range in the Tagliamento River in Italy (Bianco and Ketmaier 2005) and the Adriatic basin in Slovenia (Crivelli 2006, Bianco 2009, 2014). *Romanogobio benacensis* is considered an endangered species both globally (EN B2ab(i,ii,iii,iv,v), Crivelli 2006) and in Italy (Bianco et al. 2013).

**Figure 1.**
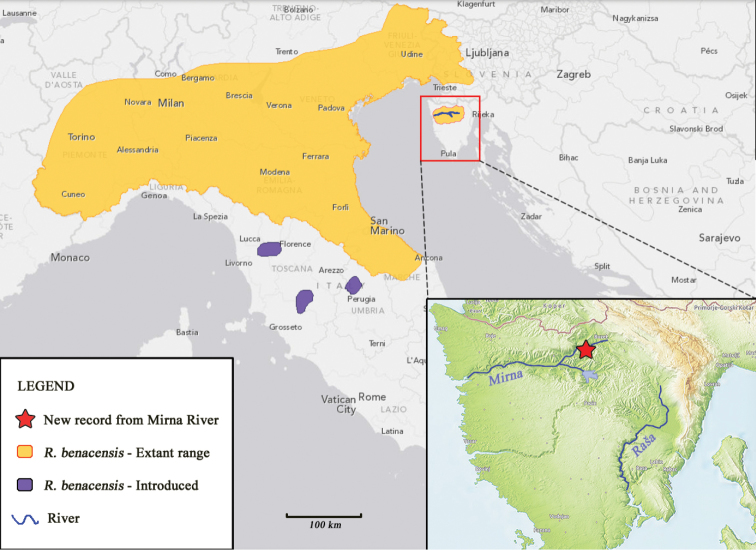
Map of distribution of *Romanogobio benacensis* in South-West Europe.

Although four species of the subfamily Gobioninae (gudgeons) have been reported in Croatia (Mustafić et al. 2005, Freyhof and Kottelat 2007, Jelić 2011a), their systematic status is uncertain (e.g., species in the *R. albipinnatus* group *sensu* Freyhof and Kottelat 2007). Mustafić et al. (2005) reported *G. gobio*, *G. uranoscopus* (Agassiz), *G. albipinnatus* Lukasch, and *G. kesslerii* Dybowski. Later, these species were assigned to *G. obtusirostris*, the Danubian longbarbel gudgeon *R. uranoscopus*, the Danubian white-finned gudgeon *R. vladykovi* (Fang), and the Kessler’s gudgeon *R. kesslerii*, respectively (Freyhof and Kottelat 2007, Jelić 2011a). In the previous studies, gudgeons in the Istra Peninsula (Mirna River; Fig. 1) were identified as *G. gobio obtusirostris* (Leiner et al. 1995) (= *G. obtusirostris*) and *G. gobio* (Mustafić et al. 2005). The main aim of this study was to investigate which gobionine species occurs in the Istra Peninsula (Croatia) and to confirm the presence of *R. benacensis* in Croatia. Gudgeon individuals collected during an ichthyological survey in the Istra Peninsula was preliminary identified as *R. benacensis* by using morphological characters (Jelić 2011a). In the present paper, samples of *R. benacensis* from different localities in Italy, Slovenia, and Croatia were described using morphological and molecular characters to support their identification in comparison with *G. obtusirostris* and *G. gobio*.

## Materials and methods

### Morphological analysis

Measurements were made according to Naseka and Freyhof (2004). All measurements were made point-to-point with a digital calliper and recorded to the nearest of 0.1 mm. Vertebrae counts are given according to Naseka (1996). Last two rays in dorsal and anal fins based on a single pterygiophore were counted as 1½ ray. Unbranched rays in dorsal and anal fins were counted from radiographs. In total, 30 morphometric indices were used for descriptions and statistical analyses as in Table 1 and 18 meristic characters as in Table 2 were examined. All characters were obtained from specimens of both sexes and combined in analyses and tables. A Mann-Whitney U Test and a Discriminant Function Analysis (DFA) were performed using STATISTICA v6.0 and PRIMER v6.1.9 to identify the most important characters that contribute to the differentiation of the two species and visualise the classification of the Reka and Mirna specimens into one of them.

**Table 1.** Morphometric characters in *Gobio gobio, Gobio obtusirostris*, and *Romanogobio benacensis*.

	*Gobio gobio*, Elba River (n = 2)	*Gobio obtusirostris*, Danube drainage (n=17)			*Gobio obtusirostris*, Reka River (n=7)			*Romanogobio benacensis*, Mirna River (n=4)			*Romanogobio benacensis*, Po and Adige drainages (n=19)		
		*range*	*M*	*SD*	*range*	*M*	*SD*	*range*	*M*	*SD*	*range*	*M*	*SD*
SL, mm	69.5–72.4	37.6–93.6	70.7		90.8–98.3	94.7	2.91	62.3–83.5	76.1		49.0–94.7	67.7	
% SL													
Body depth at dorsal-fin origin	21.6–23.5	17.9–23.8	21.0	2.01	21.2–24.7	23.3	1.04	24.4–27.7	26.0	1.53	21.7–28.3	24.7	1.65
Caudal peduncle depth	8.7–9.6	8.2–10.9	9.7	0.75	9.7–11.0	10.2	0.48	10.4–11.2	10.8	0.33	9.0–11.5	10.5	0.61
Body width at dorsal-fin origin	13.9–16.5	11.4–16.5	13.9	1.45	14.7–17.7	16.1	1.09	12.8–17.9	15.3	2.38	11.9–16.7	14.1	1.71
Width of caudal peduncle	3.0–4.0	3.0–5.9	4.0	0.75	4.2–5.6	4.7	0.52	3.5–5.0	4.3	0.64	3.3–6.5	4.5	0.79
Predorsal length	50.0–50.2	46.3–50.2	48.8	1.19	47.1–50.3	48.6	1.12	46.8–51.4	48.4	2.03	47.3–53.0	49.4	1.46
Postdorsal length	40.3–42.4	40.1–42.7	41.3	0.87	41.5–43.4	42.7	0.82	41.0–41.5	41.3	0.24	38.5–43.7	41.3	1.37
Prepelvic length	50.1–50.4	47.3–52.4	50.2	1.37	48.2–51.3	49.6	0.99	49.9–51.4	50.7	0.72	48.3–53.1	50.5	1.22
Preanal length	70.8–71.7	68.4–74.1	71.7	1.46	70.1–73.5	71.6	1.17	67.1–70.6	69.3	1.62	68.9–74.0	70.7	1.45
Distance between pectoral fin and pelvic-fin origin	24.3–24.8	21.8–27.2	24.9	1.61	24.7–26.4	25.2	0.57	24.8–30.4	27.5	2.37	21.3–28.3	25.0	1.40
Distance between pelvic fin and anal-fin origin	20.7–22.3	20.7–23.3	21.8	0.87	21.3–23.9	22.6	0.87	18.7–22.2	20.4	1.80	18.7–23.1	21.1	1.22
Distance between anus and anal-fin origin	6.3–7.1	5.1–10.1	6.8	1.21	5.8–8.0	6.6	0.72	2.5–4.5	3.4	0.88	3.1–6.2	4.4	0.90
Caudal peduncle length	20.0–22.4	17.8–22.6	20.6	1.36	18.2–22.4	20.9	1.37	19.7–23.8	21.3	1.75	18.3–22.5	20.5	1.13
Dorsal-fin length	13.1–14.3	11.9–14.3	13.3	0.81	12.1–13.6	13.0	0.53	13.8–16.3	15.0	1.26	12.1–15.8	14.3	0.93
Dorsal-fin depth	24.2–24.9	21.0–26.2	23.4	1.72	20.9–22.0	21.5	0.48	21.7–25.6	23.7	1.78	22.2–26.3	23.9	1.09
Anal-fin length	8.2–8.7	7.7–9.5	8.6	0.51	6.9–8.7	8.0	0.66	10.0–13.1	10.8	1.51	8.6–11.6	10.0	0.8
Anal-fin depth	19.0–19.4	15.8–20.8	18.4	1.50	15.8–17.2	16.5	0.45	17.9–20.5	18.9	1.10	17.3–21.6	19.1	1.2
Pectoral-fin length	20.1–22.1	18.7–23.2	21.3	1.32	18.6–21.5	19.7	1.07	21.0–22.4	21.8	0.65	19.7–24.5	21.8	1.41
Pelvic-fin length	17.1–18.3	15.8–18.3	17.2	0.92	15.9–16.4	16.1	0.20	16.0–18.5	17.5	1.07	15.8–20.3	17.8	1.04
Head length	27.7–27.8	24.4–29.9	27.4	1.82	25.6–28.1	26.7	0.91	23.9–26.1	25.2	0.99	25.0–29.1	26.9	1.11
Eye diameter	6.4–6.8	4.9–6.8	6.1	0.62	5.3–5.9	5.5	0.23	5.6–6.6	6.0	0.42	5.8–8.0	6.6	0.54
% HL													
Head depth at nape	55.9–56.7	53.3–62.5	57.5	2.61	58.5–64.1	61.6	2.12	60.0–62.1	61.0	0.88	55.8–67.1	61.7	2.52
Snout length	40.4–41.1	36.7–41.6	38.8	1.67	40.0–43.8	42.1	1.16	43.2–44.1	43.5	0.39	37.0–45.0	40.4	2.34
Eye diameter	23.1–24.4	19.1–26.2	22.8	1.84	19.2–21.6	20.7	0.90	22.8–26.2	23.9	1.51	21.0–27.9	24.6	1.6
Postorbital distance	42.1–42.6	42.1–46.8	44.7	1.64	44.3–48.7	46.3	1.58	42.3–45.4	43.8	1.67	40.7–47.5	44.4	1.78
Maximum head width	50.4–54.8	48.8–55.8	51.7	2.31	56.7–63.2	59.8	2.60	56.7–58.6	57.8	0.78	49.8–64.2	55.1	3.87
Interorbital width	27.9–30.7	26.1–34.5	29.5	2.10	32.1–36.3	34.1	1.25	31.6–35.9	33.5	1.88	27.5–34.9	30.9	1.73
Length of upper jaw	24.7–25.9	20.3–26.5	24.5	1.75	25.2–28.4	26.8	1.17	25.4–28.1	26.7	1.36	21.9–31.5	25.2	2.41
Length of lower jaw	36.0–36.1	32.2–36.1	34.9	1.22	33.1–36.1	34.4	1.10	33.1–39.9	35.3	3.13	28.8–38.5	34.5	2.51
Barbel length	26.3–27.6	24.3–34.3	27.5	2.63	21.8–28.4	25.4	2.21	30.3–39.7	35.1	4.48	24.6–42.7	33.4	4.59
Caudal peduncle depth	31.4–34.7	31.4–41.2	36.0	2.58	34.4–40.7	38.1	2.24	41.5–44.5	42.8	1.48	33.1–42.8	39.0	2.47

**Table 2.** Meristic characters in *Gobio gobio*, *Gobio obtusirostris*, and *Romanogobio benacensis*.

	*Gobio gobio*, Elba River, n=2	*Gobio obtusirostris*, Reka River, n=7			*Gobio obtusirostris*, Danube drainage, n=17 (n=53 for vertebral counts)			*Romanogobio benacensis*, Mirna River, n=4			*Romanogobio benacensis*, Po and Adige drainages, n=19		
		*range*	*Mean*	*SD*	*range*	*Mean*	*SD*	*range*	*Mean*	*SD*	*range*	*Mean*	*SD*
Unbranched dorsal-fin rays	3	3	3		3	3		4	4		3–4	3.7	0.48
Branched dorsal-fin rays	7½	7½	7½		7½	7½		7½	7½		7½	7½	
Branched anal-fin rays	6½	6½	6½		6½	6½		6½	6½		6½	6½	
Branched pectoral-fin rays	15–16	15–16	15.6	0.51	15–18	15.6	0.84	13–15	13.8	0.96	12–15	13.3	0.67
Branched pelvic-fin rays	7	7	7.0		7	7.0		7	7.0		7	7.0	
Scales in lateral row	42	41–43	41.4	0.79	40–42	41.3	0.73	38–39	38.5	0.58	37–40	38.8	0.73
Total lateral-line scales	42	41–42	41.3	0.49	39–42	41.0	1.0	37–39	38.3	0.96	37–40	38.5	0.87
Lateral-line scales to posterior margin of hypurals	39–42	38–40	39	0.58	38–40	38.9	0.81	35–36	35.8	0.50	34–37	36.4	0.80
Scales above lateral line	6	6	6.0		6	6.0		6	6.0		6	6.0	
Scales below lateral line	4	4–5	4.1	0.38	4	4.0		3–4	3.8	0.50	3–4	4.1	0.28
Scales between anus and anal-fin origin	6–9	4–7	5.4	1.27	4–7	6.1	0.71	2–3	2.3	0.50	1–5	3.7	0.85
Circumpeduncular scales	16	15–16	15.7	0.49	13–16	15.3	0.84	13–16	14.3	1.26	12–15	13.6	0.79
Predorsal scales	18–19	15–21	17.9	2.27	14–20	17.3	1.64	13–17	15.5	1.91	14–18	15.8	1.15
Total vertebrae	39–40	38–40	39.3	0.5	38–41	39.1	0.79	36–38	37.0	0.82	36–38	37.2	0.4
Abdominal vertebrae	20	20–21	20.6	0.53	20–22	20.5	0.54	19–20	19.5	0.59	18–20	19.1	0.46
Caudal vertebrae	19–20	18–19	18.7	0.49	17–20	18.5	0.68	16–18	17.5	1.0	17–18	17.9	0.32
Predorsal abdominal vertebrae	11	11	11	0.0	10–11	10.8	0.43	10–11	10.5	0.58	9–11	10.4	0.60
Preanal caudal vertebrae	2–3	1–2	1.3	0.49	1–3	1.6	0.58	0–2	1.3	0.96	1–2	1.4	0.50

(Abbreviations: SL, standard length; HL, lateral head length including skin fold; HDBI, Croatian Biological Research Society; NMW, Naturhistorisches Museum Wien; PZC, private collection of Primož Zupančič)

### Examined material

***Romanogobio benacensis.* Adriatic basin, Croatia**: HDBI 1292, 3, SL 76.8–83.5 mm, Mirna River, Kamenita Vrata, coll. D. Jelić, 19.06.2011; HDBI 1323, 1, SL 62.3 mm, Mirna River, coll. D. Jelić, 2011. **Adriatic basin, Italy**: NMW 3522–23, 2, SL 59.4–65.4 mm, Turin, coll. Steindachner, 1910; NMW 15278, 1, SL 51.3 mm, Garda Lake basin, Adige River near Rovereto; NMW 53302, 5, SL 77.2–85.6 mm, Milan, coll. Steindachner, 1864; NMW 53303, 4, SL 78.0–94.7 mm, Milan, coll. De Filippi, 08.07.1845; NMW 53304, 3, SL 67.0–68.3 mm, Italy, Garda Lake, coll. Bellotti, 1888; NMW 84845, 4, SL 33.2–67.8 mm, T. Malone, 1 km upstream on the road Rivarossa-Argentera, Torino Prov., coll. Balma, 02.1987.

***Gobio gobio*. North Sea basin, Elbe drainage**: NMW 92127, 2, SL 69.5–72.4 mm, Czech Republic, Elba River near Celakovice, coll. Oliva, 1951.

***Gobio obtusirostris.* Danube drainage**: HDBI 1331, SL 83.7 mm, Croatia, Sava system, Kupa [Kolpa] River at Ozalj, coll. D. Jelić, 2011; HDBI 1356, 3, SL 66.5–82.9 mm, Croatia, Sava drainage, Kupa River at Ozalj, coll. D. Jelić, 2011; NMW 65533, 11 (from many), SL 48.5–75.9 mm, Romania, Timis River at Urseni, coll. Bănărescu, 1963; NMW 80989, 6 (from 20), SL 71.0–75.6 mm, Austria, Raba system, Pinka River near Badersdorf, coll. Jungwirth, 1982; NMW 87485, 6, SL 75.3–88.4 mm, Slovenia, Sava system, Drtijščica tributary of Kamniška Bistrica River, coll. Krištofek, 3.4.1988; NMW 90626, 3, SL 25.6–27.0 mm, Austria, Drau River near Linz, pond near Nörsach, coll. Kofler, 02.04.1991; NMW 90825, 4, SL 77.6–99.6 mm, Slovenia, Sava system, Dobravščica and Psata tributaries of Kamniška Bistrica River, coll. Povž, 03.04.1991; NMW 90828, 3, SL 72.4–93.6 mm, Slovenia, Drava system, Rožnodolski [Pekrski potok] tributary near Maribor, coll. Povž, 3.4.1991; NMW 91507, 16 (from 20), SL 37.6–91.6 mm, Austria, Mur River downstream from Graz, coll. Schulz, 1993; **Adriatic basin, Slovenia**: PZC 677, 7, SL 90.8–98.3 mm, Reka drainage, unnamed creek at Zarečje, 45.57°N, 14.21°E, coll. Zupančič, 07.04.2007.

### DNA extraction, gene amplification, and sequencing

Besides species identification based on morphological features, species were characterised using sequences of cytochrome b gene (cytb), which is commonly used mtDNA genetic marker for species affiliation of European cyprinids (e.g. Zardoya and Doadrio 1998, Bianco and Ketmaier 2005, Perea et al. 2010). Total DNA was extracted from pectoral fin tissue with the Qiagen DNeasy Blood and Tissue Kit (Qiagen, Germany) following the manufacturer protocol. After extraction, total genomic DNA was stored on -20°C until the polymerase chain reaction (PCR) was conducted. The primers used for cytochrome b were GluF and ThrR (Machordom and Doadrio 2001). The PCR was carried out with the HotStarTaq Master Mix Kit (Qiagen). PCR reactions were prepared in a total volume of 50 µL comprised of 2.5 U HotStarTaq DNA Polymerase, 1.5 mM MgCl_2_, 200 µM each dNTP, 0.2 µM of each primer and 20 ng of DNA template. The ampliﬁcation process was conducted with the same conditions as described in Perea et al. (2010). Purification and sequencing of the PCR products were prepared by Macrogen Inc. (Seoul, South Korea) using the same primers used for gene amplification. Purified PCR products were sequenced on ABI 3730XL DNA Analyzer (Applied Biosystems, Foster City, USA). Sequence chromatograms were analysed using SEQUENCHER (version 5.3; Gene Codes Corp., Ann Arbor, USA) and aligned by eye.

The obtained sequences in this study (1141 base pairs long, bp) were examined using Nucleotide Basic Local Alignment Search Tool (Nucleotide BLAST; http://blast.ncbi.nlm.nih.gov/Blast.cgi) to screen for the most similar sequences in the GenBank nucleotide database (National Center for Biotechnology Information, U.S. National Library of Medicine, USA).

The Median-Joining (MJ) haplotype network (Bandelt et al. 1999) was used to infer the intraspecific relations in *R. benacensis* with cytb sequences obtained in this study and 342 bp sequences which were downloaded from the GenBank (Bianco and Ketmaier 2005). The MJ network was computed using PopART (Population Analysis with Reticulate Trees) v1.7 (Leigh and Bryant 2015).

Phylogenetic tree reconstructions were conducted using the cytb sequences obtained in this study and the available sequences belonging to *Gobio* and *Romanogobio* (1141 bp) from the GenBank (Briolay et al. 1998, Zardoya and Doadrio 1998, 1999, Madeira et al. 2005, Saitoh et al. 2006, Yang et al. 2006, Perea et al. 2010, Liu et al. 2010, Tang et al. 2011; Table 5). Sequences originating from the tench *Tinca tinca* (Linnaeus), the European bitterling *Rhodeus amarus* (Pallas) and the stone moroko *Pseudorasbora parva* (Temminck & Schlegel) were used as an outgroup. Newly obtained sequences in this study were deposited in the GenBank under accession numbers shown in Table 5 (will be available for publication). Phylogenetic reconstructions were inferred using three methods (Maximum Likelihood – ML, Bayesian Inference – IB and Maximum Parsimony – MP). The best-fit evolutionary model used in ML and IB was computed using jModelTest2 (version 2.1.6; Darriba et al. 2012) with the Bayesian information criterion (BIC) as implemented on the Cipres Science Gateway (version 3.1; http://www.phylo.org; Miller et al. 2010). Best-fit model of nucleotide substitution was Generalised Time Reversible (GTR) (Tavaré 1986) with a gamma distributed rate variation among sites (+G) and a significant proportion of invariant sites (+I). The ML was run using RAxML-HPC2 Workflow on XSEDE (version 8.2.8; Stamatakis 2014) on the Cipres Science Gateway with optimized parameters. For ML analysis, 200 search replicates to find the ML tree and 1000 nonparametric bootstrap replicates under the GTRGAMMA model were applied. The IB was run in MrBayes 3.2 (Ronquist et al. 2012) on the Cipres Science Gateway. Two independent runs with four MCMC chains were run for 50 million generations and sampled every 5000 generations, with temperature parameter set to 0.2 and the first 12.5 million generations discarded as burn-in. The convergence of runs was screened using AWTY (Nylander et al. 2008) while effective sample sizes of parameters were checked using TRACER 1.5 (Drummond and Rambaut 2007). The MP analysis was performed in MEGA 6.06 (Tamura et al. 2013). The MP tree was obtained using the Subtree-Pruning-Regrafting algorithm (Nei and Kumar 2000) with search level 1 in which the initial trees were obtained by the random addition of sequences (10 replicates). Nodes in phylogram which have bootstrap values P ≥ 70 in ML and MP, and posterior probabilities (pp) values ≥ 0.95 in IB were considered supported.

Three user trees (“Tree 1: *R. benacensis* sister taxon for *R. kesslerii* and *R. banaticus*”, “Tree 2: *R. benacensis* sister taxon for all *Romanogobio*”, and “Tree 3: *R. benacensis* sister taxon for all *Gobio*”) were analysed using tree topology tests [1sKH – one sided KH test based on pairwise SH tests (Shimodaira and Hasegawa 1999, Goldman et al. 2000, Kishino and Hasegawa 1989); SH – Shimodaira-Hasegawa test (2000); ELW – Expected Likelihood Weight (Strimmer-Rambaut 2002); 2sKH – two sided Kishino-Hasegawa test (1989)]. All topology tests were computed in TREE-PUZZLE v5.3rc16 (Schmidt et al. 2002).

## Results

### Comparative morphological description of *R. benacensis* from the Mirna River

**Figure 2.**
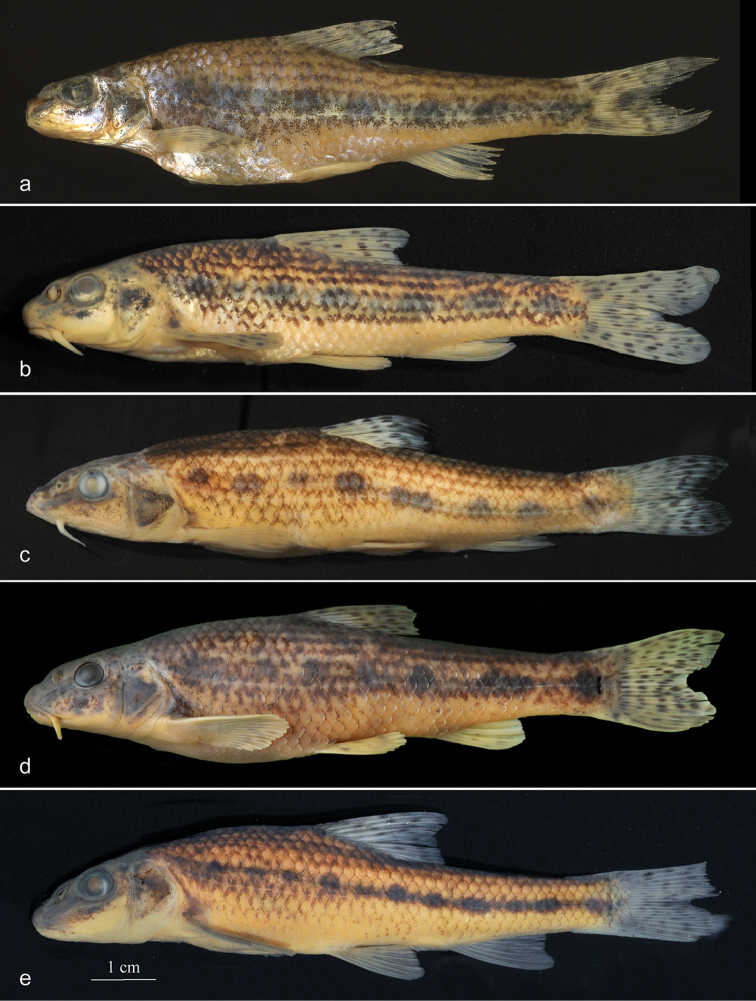
Lateral view of *Romanogobio benacensis* from the Mirna River (HDBI 1323), 62.3 mm SL (**a**) and the Po drainage (NMW 84845), 50.9 mm SL (**b**); *Gobio obtusirostris*, the Reka River (PZC), 98.3 mm SL (**c**) and the Sava River (NMW 87485), 88.4 mm SL (**d**); and *Gobio gobio*, the Elbe River (NMW 92127), 72.4 mm SL (**e**). Scale bar 1 cm.

Comparative morphological analysis of *R. benacensis* (n = 23), *G. gobio* (n = 2) and *G. obtusirostris* (n = 24) was performed based on number of available specimens. See Fig. 2a for general appearance and Table 1 for morphometric data. Below, only those characters demonstrating some difference between the species are discussed.

The body is relatively deep, the depth at the dorsal-fin origin is 24–28, averaging 26% SL (22–28, averaging 25% SL, in the Po samples) in contrast to 18–25, averaging 21–23% SL in *G. obtusirostris*.

The anus is located close to the anal-fin origin, the distance between the anus and the anal-fin origin is 3–5, averaging 3% SL (3–6, averaging 4% SL, in the Po samples) in contrast to 5–10, averaging 7% SL in *G. obtusirostris* where the anus is usually located about the midway between the pelvic and anal-fin origins (Table 2). The number of scales between the anus and the anal-fin origin is 1–5, commonly 2–4, in *R. benacensis* vs. 4–7 in *G. obtusirostris* and 6–9 in *G. gobio*.

**Figure 3.**
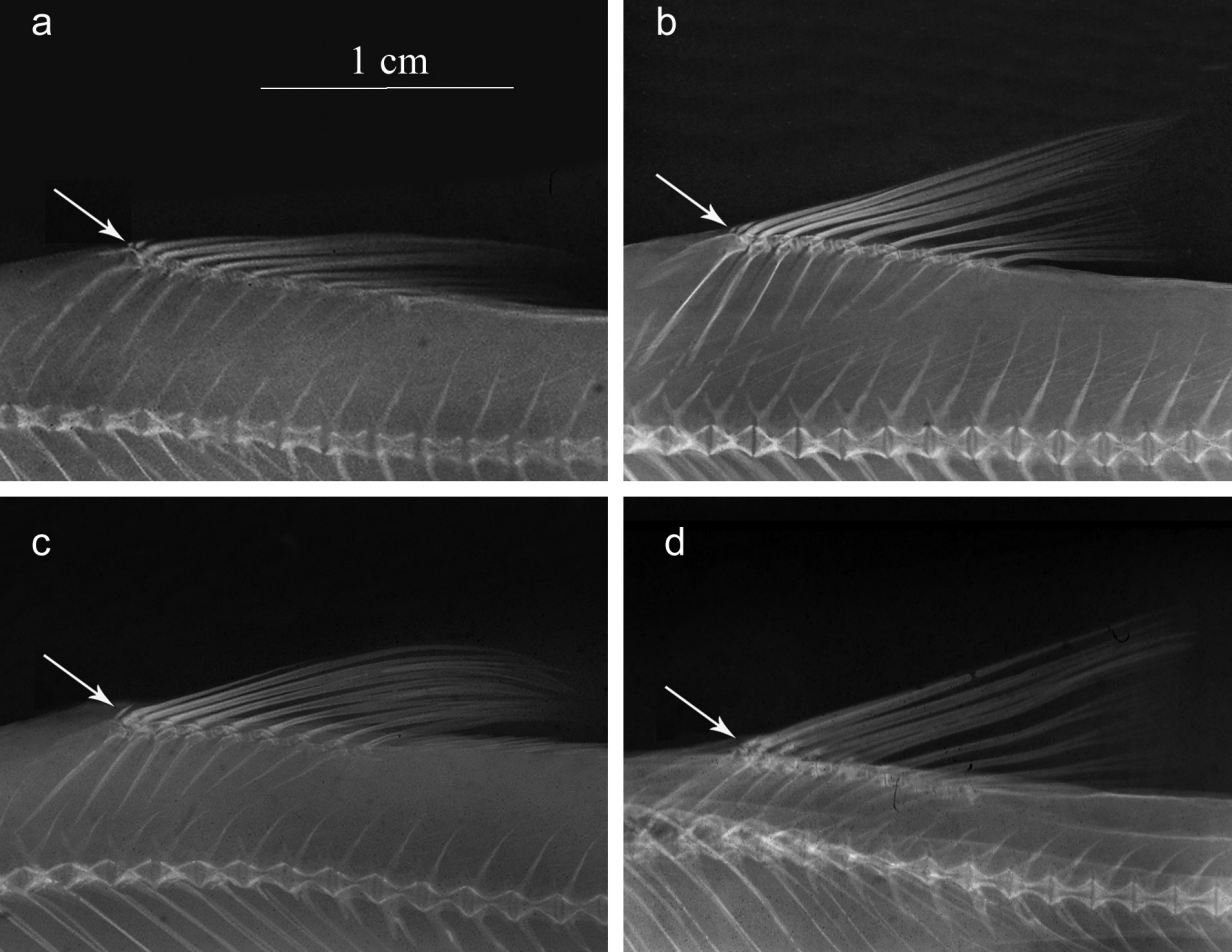
Radiographs of unbranched dorsal-fin rays. Specimens as **a–d** in Fig. 2. Arrow shows presence (**a–b**) or absence (**c–d**) of smallest anteriormost unbranched ray.

The dorsal fin has 4 unbranched rays in all specimens from Mirna and Soča rivers and in 14 (of 19) specimens from the Po drainage (Table 2, Fig. 3). This is the first known example of a species with commonly 4 unbranched dorsal-fin rays in the Gobioninae. Contrary, in *G. obtusirostris* and *G. gobio*, the number of unbranched dorsal-fin rays is always 3. In all species examined in this study the dorsal fin has 7½ branched rays and the anal fin has 3 simple and 6½ branched rays. The number of branched pectoral-fin rays is 12–15 in *R. benacensis* vs. 15–18 in *G. obtusirostris* and *G. gobio* (Table 2).

In *R. benacensis* from the Mirna River, scales along the midline of the belly extend forward to the middle of the pectoral-fin base. In *R. benacensis* from the Po drainage, scales along the midline of the belly extend forward from much behind the pectoral-fin base to the anterior end of the pectoral-fin base, commonly to the posterior end of the pectoral-fin base (Table 3). In *G. gobio*, the breast and throat are more scaled; scales along the midline of the belly extend forward from the middle of the pectoral-fin base to a point in front of the anterior end of the pectoral-fin base. In *G. obtusirostris*, the throat and breast are less scaled similarly to *R. benacensis*; scales along the midline of the belly extend forward from a point much behind the pectoral-fin base to the anterior end of the pectoral-fin base (Table 3).

**Table 3.** Number of specimens of *Gobio gobio*, *Gobio obtusirostris*, and *Romanogobio benacensis* showing character states of the development (presence) of scales on the ventral side (throat and breast). Each character state refers to the anteriormost scale along the ventral midline.

Species and locality	In front of pectoral-fin base	Anterior end of pectoral-fin base	Middle of pectoral-fin base	Posterior end of pectoral-fin base	Behind pectoral-fin base
*Gobio gobio*, Elba River	1		1		
*Gobio obtusirostris*, Reka River		1		3	3
*Gobio obtusirostris*, Danube drainage		1		13	3
*Romanogobio benacensis*, Mirna River			4		
*Romanogobio benacensis*, Po drainage		1	4	10	4

The lateral line is complete, with 37–39 total scales averaging 38.6 (36–40 averaging 38.8 in the Po samples). These counts are lower than in *G. gobio* and *G. obtusirostris* which have a range of 39–42 scales, and averages of 42.0 and 41.3, respectively. Other scale counts can be also seen in Table 2. No epithelial keels on scales were found in specimens of *R. benacensis*.

The barbel is reaching the vertical through the middle of the pupil to the posterior margin of the eye (Table 4). Similar character states are also typical to *R. benacensis* from the Po drainage: the barbel is reaching the vertical through the middle of the pupil to behind the posterior margin of the eye, more frequently between the posterior margin of the pupil and the posterior margin of the eye. On average, the barbel is longer in *R. benacensis* than in *G. gobio* and *G. obtusirostris* (30–39% HL, averaging 35 in *R. benacensis* from the Mirna River and 26–37% HL, averaging 32.5 in *R. benacensis* from the Po drainage vs. 22–28% HL, averaging 25 in *G. obtusirostris* from the Reka River and 24–34% HL, averaging 27.5 in *G. obtusirostris* from the Danube drainage) (Table 1). In *G. obtusirostris*, the barbel is commonly reaching the vertical through the anterior margin of the pupil to the posterior margin of the pupil (Table 4).

**Table 4.** Character states of the position of the posteriormost extremity of the barbel in *Gobio gobio, Gobio obtusirostris*, and *Romanogobio benacensis*.

Species and locality	Barbel reaching to vertical of:					
	anterior margin of pupil	middle of pupil	posterior margin of pupil	between pupil and posterior margin of eye	posterior margin of eye	behind posterior margin of eye
*Gobio gobio*, Elba River		2				
*Gobio obtusirostris*, Reka River	4	1	2			
*Gobio obtusirostris*, Danube drainage	3	6	2	6		
*Romanogobio benacensis*, Mirna River		1	2		1	
*Romanogobio benacensis*, Po drainage		4	4	8	1	1

Total vertebrae are 36–38, 19–20 abdominal, and (16)18 caudal including 0–2 preanal. 10–11 predorsal vertebrae. The vertebral counts in the Mirna samples of *R. benacensis* are similar to those in the Po samples, the most frequent vertebral formulae are 19+18 (17), 20+17 (2), and 20+18 (2) (Table 2). *G. gobio* and *G. obtusirostris* differ by higher average numbers of total, abdominal, and caudal numbers, the most frequent vertebral formulae are 21+18 (18), 20+19 (15), 20+18 (9), and 21+19 (8).

Laterally (examined on both sides) with 7–8, usually 7 roundish dark blotches. The size of a blotch varies but it is relatively large: the size of the blotch below the dorsal-fin origin is about (close to) or larger than the horizontal eye diameter. The same pattern of the blotches, 5–8, usually 6 or 7, is found in the examined specimens from the Po and Soča drainages. In *G. gobio* and *G. obtusirostris*, the blotches are smaller and more numerous, 7–11, usually 8 or 9, and the size of the blotch below the dorsal-fin origin is about the half horizontal eye diameter (Fig. 2).

**Table 5.** List of species used for phylogenetic tree inference on cytb sequences. Data on catalogue numbers of analysed specimens, localities, GenBank Accession numbers, sequences lengths, and references are shown.

Species	Catalogue no.	Locality	Accession No.	Sequence length (bp)	Reference
* Romanogobio benacensis *	HDBI 1323/tissue ID 771	Croatia: Mirna River, Kamenita Vrata	xxx	1141	This study
* Romanogobio benacensis *	HDBI 1292/tissue ID 772	Croatia: Mirna River, Kamenita Vrata	xxx	1141	This study
* Romanogobio benacensis *	HDBI 1292/tissue ID 773	Croatia: Mirna River, Kamenita Vrata	xxx	1141	This study
* Romanogobio benacensis *	HDBI 1292/tissue ID 774	Croatia: Mirna River, Kamenita Vrata	xxx	1141	This study
* Gobio obtusirostris *	HDBI/tissue ID 775	Bosnia and Herzegovina: Boračko Lake	xxx	1141	This study
* Gobio gobio *	MEL	Italy: Meletta River	AY641521	342	Bianco and Ketmaier (2005)
* Romanogobio benacensis *	TAG	Italy: Tagliamento River	AY641522	342	Bianco and Ketmaier (2005)
* Gobio gobio *	ASS	Italy: Assino River	AY641523	342	Bianco and Ketmaier (2005)
* Romanogobio benacensis *	OMB	Italy: Ombrone River	AY641524	342	Bianco and Ketmaier (2005)
* Gobio gobio *	BAD	Italy: Badolato River	AY641525	342	Bianco and Ketmaier (2005)
* Gobio gobio *		France: Rhone River	Y10452	1141	Briolay et al. (1998)
*Gobio lozanoi* Doadrio and Madeira, 2004		Spain: Tajo River	AF045996	1141	Zardoya and Doadrio (1998)
* Gobio obtusirostris *		Greece: Gallikos River	AF090750	1141	Zardoya and Doadrio (1999)
*Romanogobio banarescui* (Dimovski and Grupche, 1974)		Greece: Aliakmon River	AF090751	1141	Zardoya and Doadrio (1999)
*Romanogobio ciscaucasicus* (Berg, 1932)	Gobio_ciscaucasicus	Russia: Kuma River	AF095607	1141	Zardoya and Doadrio (1999)
* Romanogobio uranoscopus *	G.ura.34	Romania: Valsan River/Valsanesti	AY426593	1141	Madeira et al. (2005)
* Gobio lozanoi *	G.go.13FR.ADOUR	France: Adour River	AY426572	1141	Madeira et al. (2005)
* Gobio gobio *	G.go.33Czech.R	Czech Republic	AY426592	1141	Madeira et al. (2005)
* Gobio gobio *		Czech Republic: Plana	AB239596	1141	Saitoh et al. (2006)
* Gobio gobio *	Gobio_gobio (2)	Germany: Rhine River	AY953007	1141	Yang et al. (2006)
* Gobio obtusirostris *	Gobio_gobio (1)	Romania	EF173619	1141	Luca et al. (direct submission)
* Gobio obtusirostris *	MNCN_AT4759	Slovenia: Sevnica River	HM560092	1141	Perea et al. (2010)
* Romanogobio kesslerii *	WL*0653a	Ukraine: middle Dniestr River close to type locality	AY952328	1141	Witte (direct submission)
*Romanogobio banaticus* (Bănărescu, 1960)	WL*0626a	Romania: middle Nera River (Danube drainage)	AY952329	1141	Witte (direct submission)
* Romanogobio banaticus *	WL*0626b	Romania: middle Nera River (Danube drainage)	AY952330	1141	Witte (direct submission)
* Romanogobio uranoscopus *	WL*0624a	Romania: middle Nera River (Danube drainage)	AY952331	1141	Witte (direct submission)
*Romanogobio macropterus* (Kamensky, 1901)	WL*0628a	Turkey: Aras River	AY952332	1141	Witte (direct submission)
*Gobio macrocephalus* Mori, 1930	Gobio_macrocephalus	China: Yanji, Tumenjiang River	AY953006	1141	Yang et al. (2006)
*Gobio cynocephalus* Dybowski, 1869	Gobio_cynocephalus	China: Fuyuan, Amur River	AY953005	1141	Yang et al. (2006)
*Romanogobio tenuicorpus* (Mori, 1934)	Romanogobio_tenuicorpus	China: Yellow River	AY953004	1141	Yang et al. (2006)
*Gobio huanghensis* Luo, Le and Chen, 1977	Gobio_huanghensis	China	FJ904648	1141	Qi et al. (direct submission)
*Gobio soldatovi* Berg, 1914	IHCAS:0210055	China: Kaiyuan, Liahe River	EU934491	1141	Liu et al. (2010)
* Romanogobio tenuicorpus *	CTOL00130	n/a	JN003327	1141	Tang et al. (2011)
* Romanogobio ciscaucasicus *	CTOL00128	n/a	JN003325	1141	Tang et al. (2011)
*Romanogobio tanaiticus* Naseka, 2001	CTOL00129	n/a	JN003324	1141	Tang et al. (2011)
*Gobio cynocephalus* Dybowski, 1869	CTOL00112	n/a	JN003328	1141	Tang et al. (2011)
*Gobio coriparoides* Nichols, 1925	CTOL00375	n/a	JN003326	1141	Tang et al. (2011)
* Tinca tinca *	* Tinca tinca *	Bosnia and Herzegovina: Trebišnjica River, Ravno	HM560230	1141	Perea et al. (2010)
* Rhodeus amarus *	* Rhodeus amarus *	Czech Republic: Libechovka River, Elbe River	HM560156	1141	Perea et al. (2010)
* Pseudorasbora parva *	* Pseudorasbora parva *	Turkey: Kizilirmak River, Kirsehir	HM560155	1141	Perea et al. (2010)

### Statistical analysis

A Mann-Whitney U test revealed seven morphometric and nine meristic characters different on a statistically significant (0.01%) level between the samples of typical *G. obtusirostris* (Danube specimens) and typical *R. benacensis* (Po and Adige specimens): the body depth at the dorsal-fin origin, the distance between the pelvic fin and the anal-fin origin, the distance between the anus and the anal-fin origin, the dorsal-fin length, the anal-fin length, the the interorbital width, the barbel length, the number of unbranched dorsal-fin rays, the number of branched pectoral-fin rays, the numbers of scales in lateral series, total lateral-line scales and lateral-line scales to the posterior margin of hypurals, the number of circumpeduncular scales, the number of scales between the anus and the anal-fin origin, and the numbers of total and abdominal vertebrae.

**Figure 5.**
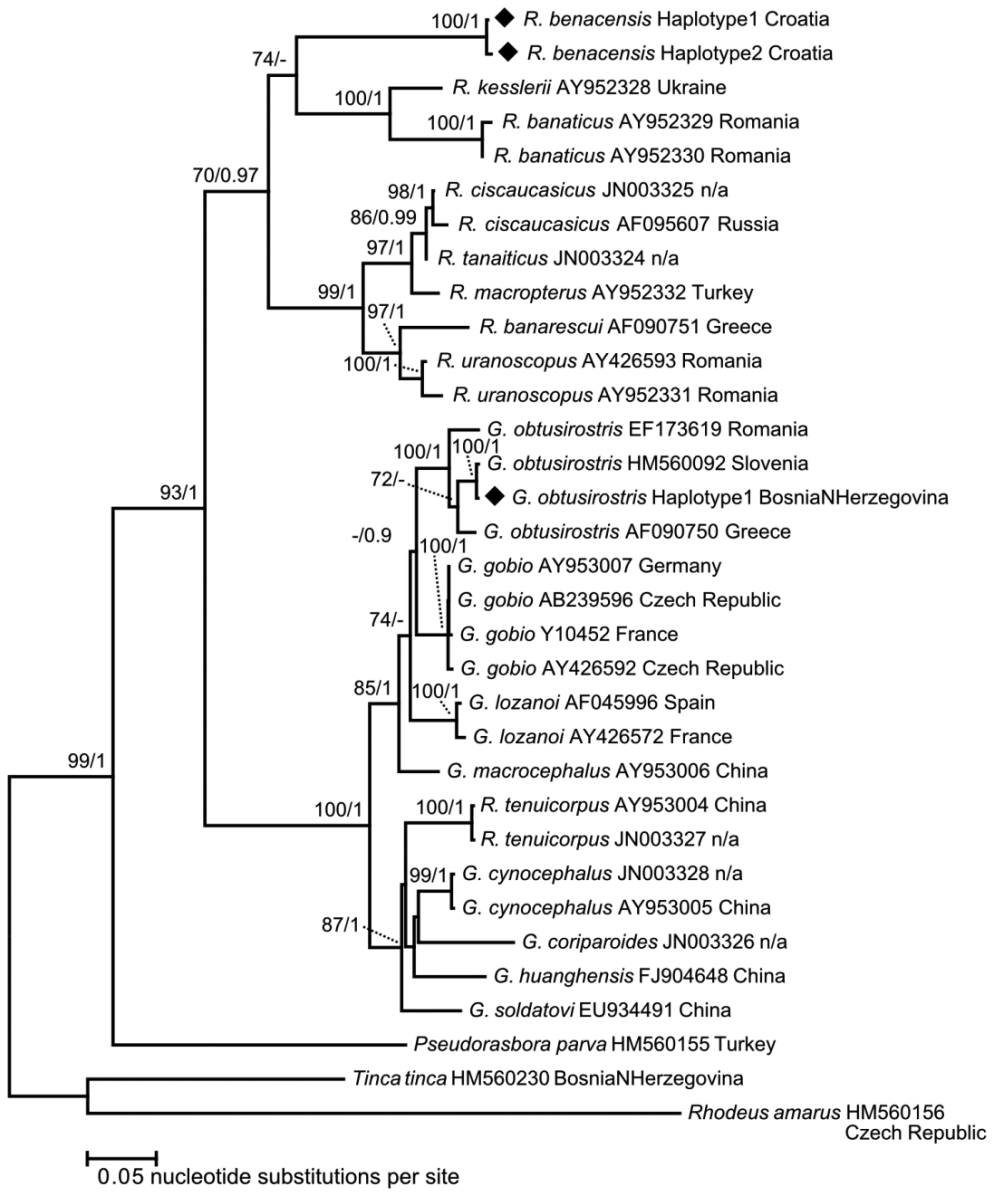
Result of DFA performed on 15 (8 meristic and 7 morphometric) distinguishing characters to classify Reka and Mirna samples.

These 16 distinguishing characters were used for a DFA in order to classify the Reka and Mirna samples into one of the two species. DFA statistics values are as follows: Wilks’ Lambda 0.00721, approx. F (45, 78) =7.3894, p<0.0000. The Mirna specimens are the closest to Italian *R. benacensis* (Fig. 5) (Squared Mahalanobis Distance equals 21.5024 vs. 74.7999 between *G. obtusirostris* from the Danube and Italian *R. benacensis*).

### Phylogenetic tree inference

**Figure 6.**
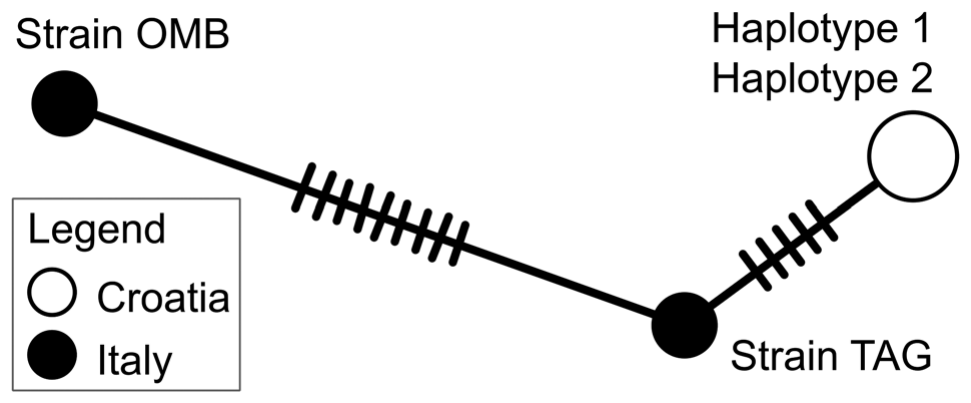
The MJ haplotype network of *Romanogobio benacensis*

Two unique cytb haplotypes were detected in four specimens from the Mirna River (Table 5) Haplotype 1 originates from three specimens (HDBI 1323/tissue 771, HDBI 1292/tissue ID 772, and HDBI 1292/tissue ID 773) whereas Haplotype 2 was observed in one specimen (HDBI 1292/tissue ID 774). Haplotype 1 and Haplotype 2 have 99% similarity score (1136 identical nucleotide positions in 1141 bp sequence alignment). Nucleotide BLAST search using Haplotype 1 resulted in 99% similarity score (337 identical nucleotide positions in 342 bp alignment) with GenBank entry AY641522 designated as “strain TAG” of *G. benacensis* in Bianco and Ketmaier (2005), validating morphological determination of *R. benacensis* in this study. The second top match in BLAST search using Haplotype 1 was GenBank entry AY641524 designated as “strain OMB” of *G. benacensis* in Bianco and Ketmaier (2005) with similarity score 328/342 (96%). The 342 bp sequence alignment of *R. benacensis* were analysed with the MJ haplotype network (Fig. 6). Haplotypes 1 and 2 from Croatia differ by five mutational steps from the “strain TAG” and can be considered as members of this strain. There are nine mutational steps between “strain TAG” and “strain OMB”, confirming there are two strains in *R. benacensis* (Bianco and Ketmaier (2005). The sequences obtained in Bianco and Ketmaier (2005) (342 bp) were not used in further phylogenetic tree reconstruction in this study to avoid inclusion of significant proportion of missing sites in the final sequence alignment (1141 bp). The ML, IB and MP provided congruent trees with no supported contradictions (Fig. 7). Results of phylogenetic reconstruction indicated *Romanogobio* and *Gobio* as two statistically supported clades (P (ML) = 70, pp (IB) = 0.97, P (MP) = 91, and P (ML) = 100, pp (IB) = 1, P (MP) = 100, respectively). High statistical support was also observed for the node showing divergence between these two clades (P (ML) = 93, pp (IB) = 1, P (MP) = 80). Results of phylogenetic inference showed that *R. benacensis* belongs to the clade of *Romanogobio* (Fig. 7).

All topology tests (Table 6) indicated that best topology is presented in “Tree 1: *R. benacensis* sister taxon for *R. kesslerii* and *R. banaticus*” vs. “Tree 2: *R. benacensis* sister taxon for all *Romanogobio*” and “Tree 3: *R. benacensis* sister taxon for all *Gobio*”.

**Table 6.** Comparison of user trees (“Tree 1: *Romanogobio benacensis* sister taxon for *Romanogobio kesslerii* and *Romanogobio banaticus*”, “Tree 2: *R. benacensis* sister taxon for all *Romanogobio*”, and “Tree 3: *R. benacensis* sister taxon for all *Gobio*”). The columns show the results and p-values of the following tests: 1sKH - one sided KH test based on pairwise SH tests (Shimodaira and Hasegawa 1999, Goldman et al., 2000, Kishino and Hasegawa 1989); SH - Shimodaira-Hasegawa test (1999); ELW - Expected Likelihood Weight (Strimmer and Rambaut 2002); 2sKH - two sided Kishino-Hasegawa test (1989). Plus signs denote the confidence sets. Minus signs denote significant exclusion. All tests used 5% significance level. 1sKH, SH, and ELW performed 1000 resamplings using the RELL method. 1sKH and 2sKH are correct to the 2nd position after the the decimal point of the log-likelihoods.

Tree	log L	difference	S.E.	p-1sKH	p-SH	c-ELW	2sKH
1	-8489.12	0.00	<---- best	1.0000 +	1.0000 +	0.7078 +	best
2	-8491.32	2.20	2.8840	0.2040 +	0.5230 +	0.2036 +	+
3	-8499.39	10.27	8.1588	0.1010 +	0.1250 +	0.0887 +	+

## Discussion

Morphological data in this study confirm observations of the previous authors (Bianco and Taraborelli 1984, Bianco and Ketmaier 2005, Kottelat and Freyhof 2007) that *R. benacensis* differs from *G. gobio* and *G. obtusirostris* by a shorter distance between the anus and the anal-fin origin. However, this character is not completely discriminating in this study – there are specimens of both *R. benacensis* (including all examined specimens from the Soča River) and *Gobio* with 4 or 5 scales between the anus and the anal-fin origin. However, the length of the distance between the anus and the anal-fin origin is still diagnostic: in *R. benacensis*, the distance between the anus and the anal-fin origin (3–5% SL) is smaller than the eye diameter (6–8% SL) while in *G. gobio* and *G. obtusirostris* this distance (5−10% SL) is equal or larger than the eye diameter (5–7% SL). Kottelat and Freyhof (2007) also stated a difference in the scale pattern on the abdomen in *R. benacensis*; the scales extend only to a point between the pectoral and pelvic-fin bases vs. a level of the posterior end of the pectoral-fin base in *Gobio*. However, our data (Table 3) did not confirm this character to be clearly diagnostic for the two taxa.

*Romanogobio benacensis* also differs from *G. gobio* and *G. obtusirostris* by a number of character states which includes often four (vs. three) unbranched dorsal-fin rays, lower numbers of branched pectoral-fin rays (12–15 vs. 15–18), lateral-line scales (total number 37–40 vs. 39–42), total vertebrae (36–38 vs. 38–41), abdominal vertebrae (18–20 vs. 20–22), and caudal vertebrae (16–18 vs. 17–20) (Table 2).

**Figure 4.**
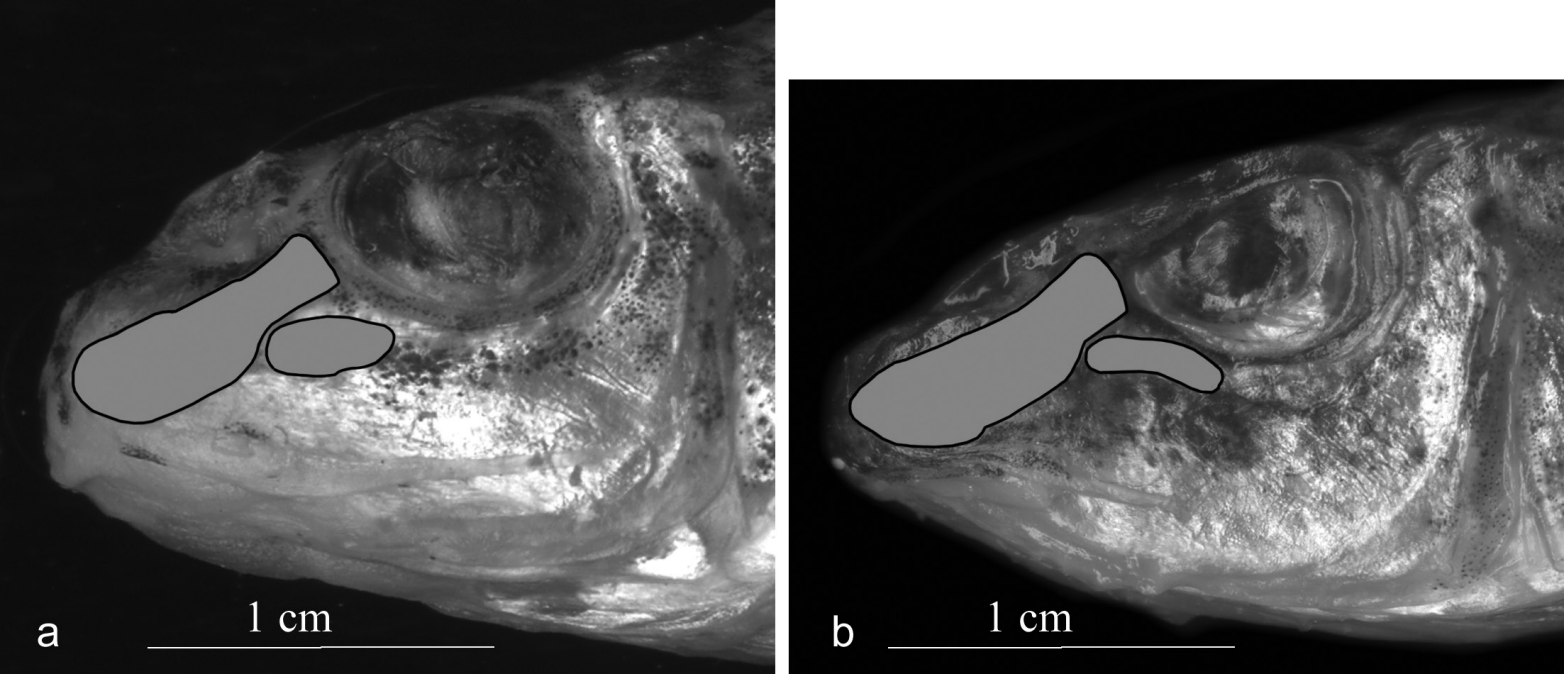
Lateral view of the head of *Romanogobio benacensis* (**a**) and *Gobio obtusirostris* (**b**); the 1^st^ and 2^nd^ infraorbital bones are shaded. Scale bar 1 cm.

Some of the morphological characters of *R. benacensis* do correspond to those diagnostic of the genus *Gobio*. As shown by Kottelat and Freyhof (2007), *R. benacensis* does not have epithelial crests on scales on the dorsal surface of the body, a shallow caudal peduncle, and a long distance between the anus and the anal-fin origin, the characters typical for *Romanogobio.* As to the position of the anus (in relation to anal-fin base), it is closer to the anal fin in *R. benacensis* than even in *Gobio*; a position of the anus at (or close to) the anal-fin origin is a plesiomorphic feature in the Gobioninae (Naseka 1996). The genus *Romanogobio* also differs from *Gobio* in having the supraethmoid wide (wider than long) vs. elongated (longer than wide); a high and oval second infraorbital (Fig. 4a) vs. narrow and rod-shaped (Fig. 4b), and a relatively high number of vertebrae both total and in the regions (the modal vertebral formula 42:(11)20(5)+(3)21(18) vs. 39:(11)21 (5)+(1)18(17) (Naseka 1996, Naseka et al. 2002). As shown above, *R. benacensis* possess low vertebral counts which are even lower than those found in the examined *Gobio* species. But the shape of the supraethmoid and infraorbitals are similar to that typical of *Romanogobio*.

As shown above in the results of the morphological analysis, the examined sample from the Reka River is *G. obtusirostris*, not *R. benacensis*. This was not expected having in mind the current hydrological features of the Reka River which drains into the Adriatic Sea. The Reka River (Notranjska Reka) originates in Croatia and flows 54 km through western Slovenia, disappears in the Škocjanske jame underground cave system and reappears again after 38 km as a part of the Timavo River in Italy flowing into the Adriatic Sea. Although results in this study indicates that *R. benacensis* is not present in the Reka River, further analyses using more specimens are needed for a final systematic conclusions. Also, the native status of *G. obtusirostris* in the Reka River is unclear. It could be a non-native species similar to an introduced chub *Squalius cephalus* (Linnaeus).

The morphological data discussed above confirmed that gudgeons from the Mirna River can be assigned to *R. benacensis*.

**Figure 7.**
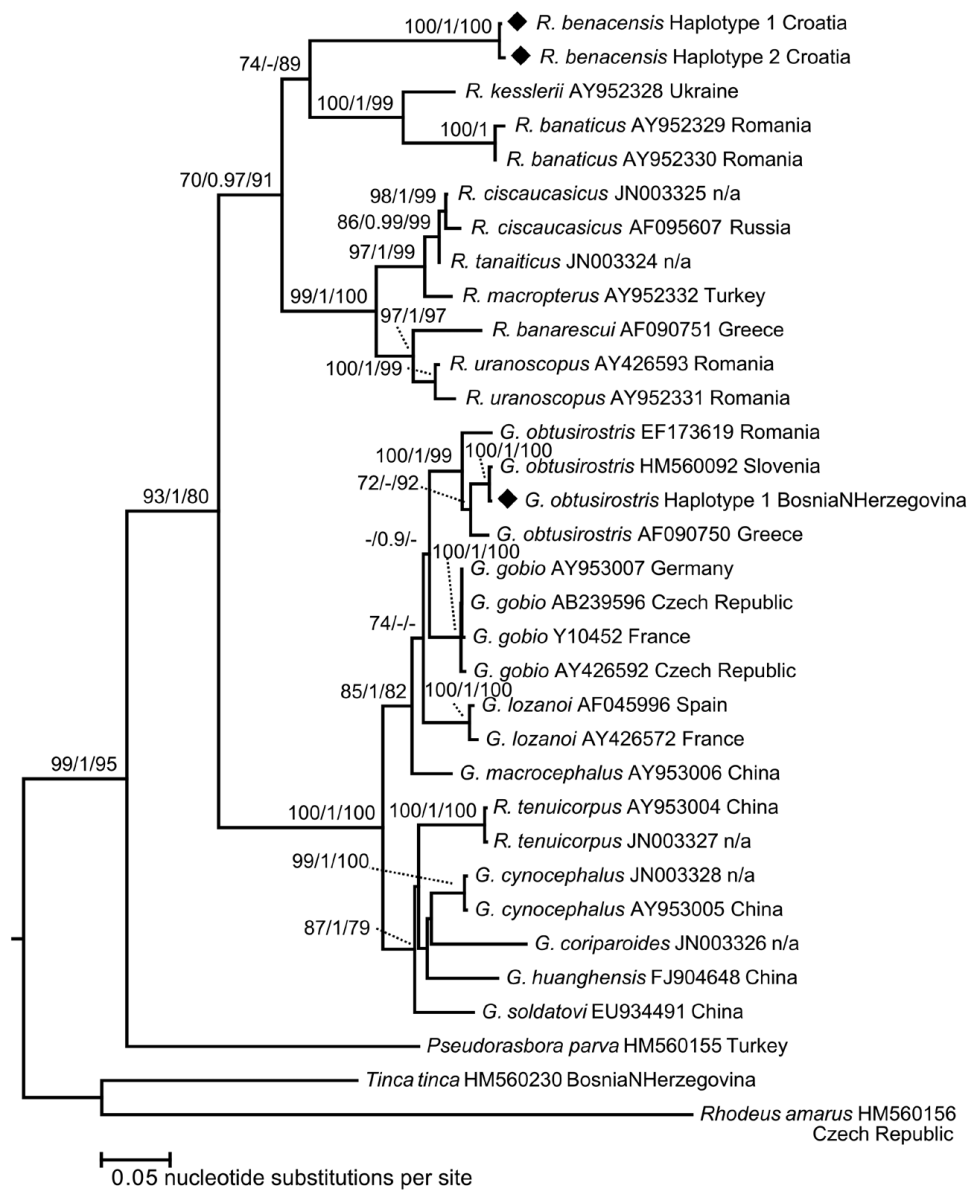
Phylogenetic tree inferred by ML analysis using cytb sequences of *Romanogobio* and *Gobio*. Newly obtained haplotypes in this study were marked by black rhombi. Node supports are given as bootstrap values (P) in ML and MP analyses (showing values ≥ 70) and posterior probabilities (pp) in IB (showing values ≥ 0.9).

Phylogenetic reconstruction in this study (Fig. 7) indicated *Gobio* and *Romanogobio* as two statistically supported clades; with *R. benacensis* as a member of *Romanogobio* clade. Although results of Bianco and Ketmaier (2005) and Geiger et al. (2012) questioned the phylogenetic recognition of *Romanogobio* as a distinct clade in respect to *Gobio*, the monophyletic status of *Romanogobio* was shown by Madeira et al. (2005) and in comprehensive studies on Gobionine phylogeny (Yang et al. 2006, Tang et al. 2011). Difference in statistical supports for clades *Romanogobio* and *Gobio* among studies in which cytb was applied (Bianco and Ketmaier 2005, Madeira et al. 2005, Yang et al. 2006, Tang et al. 2011, this study) most likely originates from the use of different sequence lengths (342 bp in Bianco and Ketmaier 2005 vs. 1141 bp in other studies). Similarly, a 657 bp COI fragment used in Geiger et al. (2012) could be less informative than a longer cytb fragment used in this study. A better resolution is expected using longer sequences in phylogenetic reconstructions although non-hierarchical relations can characterize some phylogenies no matter of the length of used sequences (Strimmer and von Haeseler 1997).

The Mirna population represents a single area of occurrence of *R. benacensis* out of its known distribution range in the south-east of the Soča (Isonzo) drainage. If the species is native in the Mirna River, this is the only occurrence of a native species belonging to the genus *Romanogobio* and the subfamily Gobioninae along the Croatian section of the Adriatic coast (the Dalmatia freshwater ecoregion *sensu* Abell et al. 2008). It may be an evidence of past connections between Istrian rivers and the paleo-Po drainage. A similar “paleo-Po” distribution is reported for the triotto *Leucos aula* (Bonaparte), the Padanian barbel *Barbus plebejus* Bonaparte, the alborella *Alburnus arborella* (Bonaparte) and some other fish species (Kottelat and Freyhof 2007), also for an amphibian, the Italian agile frog *Rana latastei* Boulenger (Gasc et al. 1997) and a freshwater decapod crustacean, the white-clawed crayfish *Austropotamobius pallipes* (Lereboullet) (Jelić et al. 2016b). However, a human mediated translocation of *R. benacensis* cannot be excluded; rather it is a supposition to be further investigated. For example, *G. obtusirostris* in the Ričica River (Lika Region, Adriatic basin) probably originates from the Danube drainage (Jelić et al. 2016a).

Only four specimens of *R. benacensis* were collected in spite of an intensive sampling effort. Since no other gudgeon species was reported in the Mirna River, an ongoing population extirpation by competition (e.g. with *G. gobio* as reported by Bianco and Ketmaier (2005) in Italy) can be excluded as a reason for low population density. Nevertheless, competition with non-native cyprinid species such as *P. parva*, or a predation by allochthonous piscivorous fish such as the pike *Esox lucius* (Linnaeus) or the pike-perch *Sander lucioperca* (Linnaeus) should not be excluded.

Therefore, having in mind that small-sized populations are more prone to extirpation due to genetic drift, extinction vortex, etc., it is necessary to implement systematical monitoring on the present *R. benacensis* population, accompanied with a more intense sampling in order to reveal possible remaining populations and to characterize the gene pool of this endangered species, both crucial issues for further management and conservation.

***Vernacular name***. *Romanogobio benacensis* does not have any Croatian name as it has been only recently discovered in Croatian national territory. We offer “Talijanska krkuša” as its Croatian name, which originate from translation of vernacular name on English (the Italian gudgeon) and Italian (il gobione Italiano).
